# Cross-model validation of public health microsimulation models; comparing two models on estimated effects of a weight management intervention

**DOI:** 10.1186/s12889-024-18134-4

**Published:** 2024-03-12

**Authors:** Sarah Bates, Penny Breeze, Chloe Thomas, Christopher Jackson, Oliver Church, Alan Brennan

**Affiliations:** 1 University of Sheffield, Sheffield, UK; 2 University of Cambridge, Cambridge, UK

**Keywords:** Cross-model comparison, Health economic modelling, Weight management

## Abstract

**Background:**

Health economic modelling indicates that referral to a behavioural weight management programme is cost saving and generates QALY gains compared with a brief intervention. The aim of this study was to conduct a cross-model validation comparing outcomes from this cost-effectiveness analysis to those of a comparator model, to understand how differences in model structure contribute to outcomes.

**Methods:**

The outcomes produced by two models, the School for Public Health Research diabetes prevention (SPHR) and Health Checks (HC) models, were compared for three weight-management programme strategies; Weight Watchers (WW) for 12 weeks, WW for 52 weeks, and a brief intervention, and a simulated no intervention scenario. Model inputs were standardised, and iterative adjustments were made to each model to identify drivers of differences in key outcomes.

**Results:**

The total QALYs estimated by the HC model were higher in all treatment groups than those estimated by the SPHR model, and there was a large difference in incremental QALYs between the models. SPHR simulated greater QALY gains for 12-week WW and 52-week WW relative to the Brief Intervention. Comparisons across socioeconomic groups found a stronger socioeconomic gradient in the SPHR model. Removing the impact of treatment on HbA1c from the SPHR model, running both models only with the conditions that the models have in common and, to a lesser extent, changing the data used to estimate risk factor trajectories, resulted in more consistent model outcomes.

**Conclusions:**

The key driver of difference between the models was the inclusion of extra evidence-based detail in SPHR on the impacts of treatments on HbA1c. The conclusions were less sensitive to the dataset used to inform the risk factor trajectories. These findings strengthen the original cost-effectiveness analyses of the weight management interventions and provide an increased understanding of what is structurally important in the models.

**Supplementary Information:**

The online version contains supplementary material available at 10.1186/s12889-024-18134-4.

## Background

Behavioural weight management programmes have been shown to result in weight loss and reductions in glycaemia [1–3]. The WRAP trial showed that referral to a commercial open-group behavioural programme (WW, formerly Weight Watchers) for 12 weeks or 52 weeks resulted in greater weight loss during a 2-year follow-up than a brief intervention (a booklet of self-help weight-management strategies) [1]. At the five-year follow-up of the trial, there was no significant difference in weight between the groups, but there was some weight loss maintenance [4]. Health economic modelling over the lifetime estimated that the 12-week and 52-week programs were cost saving and generated QALY gains compared with the brief intervention. Relative to natural history, the 52-week programme generated greater benefits than the 12-week programme. To improve model credibility and align with the International Society for Pharmacoeconomics and Outcomes Research–Society for Medical Decision Making (ISPOR-SMDM) international best practice recommendations, cross-model validation is needed [5].

Cross-model validation, also known as cross validation, comparative modelling or convergent validation, is the process of simulating the same decision problem with two or more models and comparing the predictions and outcomes [6] and is recommended by modelling good research practices and guidelines [5, 7, 8] Existing guidance is summarised in Supplementary Material, Table S1. Although it cannot be used as evidence that the model predicts outcomes accurately, it can give more confidence in the outcomes and credibility to the model if different models result in similar outcomes or the same decision. To maximise the value of the cross-model validation, models should be developed independently, and modellers should collaborate in the comparison process [5]. Cross-model validation is particularly important for validating the long-term impact of an intervention. External validation (comparisons to historical event data) is a useful form of validation for health trajectories estimated in the absence of an intervention (i.e., standard care). However, novel intervention outcomes cannot be compared with historical data. Furthermore, it can be challenging to find, and access, representative datasets to validate model outcomes against, particularly for utilities (a measure of the preference or value assigned to a particular health state), quality-adjusted life years (QALYs) and aggregated costs. Cross-model validation of the long-term impact of the treatment is likely to be particularly important for public health interventions in which the long-term (and even short-term) impacts of an intervention on health are unknown [9].

Although cross-model validation is recommended, there is no clear guidance on how to carry out the validation and there is variation in how it is conducted [10]. Few studies provide a detailed explanation of the validation framework used, and there was variation in reporting [6]. Examples of cross-model validation most commonly include a comparison of model outcomes against previously published results [11]. In many published cross-model validations, individual teams run scenarios on their models, and the results are compared [8, 12–16]. Although this enables many models to be compared at once with multiple scenarios, it can be more difficult to establish how model structure impacts these differences. Each team has a less detailed understanding of other models, and the process requires extensive time commitment from all participants, which can act as a barrier to engagement. In one example, the authors had access to both models that had been developed independently [6]. Expansion in open-source modelling provides opportunities to develop cross-model validation processes to benefit decision-makers and modellers.

Comparisons of public health models that estimate the impact of behavioural interventions are challenging. When comparing public health models, a single intervention may impact several risk factors and the risk of many different health outcomes, which may result in large differences between model structures and assumptions even when examining a single health behaviour (e.g., eating healthily) [9]. Furthermore, microsimulation models are often used for evaluating behavioural interventions because they allow flexibility in structure and therefore can be used to represent the various pathways from intervention to risk factors to outcomes. This may contribute to greater variation between model structures. Observations from a cross-model validation of public health interventions suggest that there are large variations in costs per QALY between models [10]. Differences in model structure are likely to make differences in model outcomes difficult to interpret and risk reducing trust in findings for decision-makers. This highlights the need for publicly available detailed cross-model validations of public health models; it enables greater understanding of the impact of model structure on outcomes for the decision problem of interest but also because it can inform the development of existing and new models.

The aim of this study was to conduct a cross-model validation to compare estimations of long-term effectiveness generated by the SPHR model, as part of an existing cost-effectiveness analysis, with those of a comparator model that also quantified long-term effectiveness of weight management, to help understand how differences in model structure contribute to outcomes.

## Method

### Decision problem

The extended and standard duration weight-loss programme referrals for adults in primary care (WRAP) trial was a randomised controlled trial in which participants were assigned to a brief intervention (brief advice and self-help materials) or a 12- or 52-week weight-management programme (WW) [4]. The 5-year follow-up was registered with Current Controlled Trials (ISRCTN64986150) on 01/02/2018. Participants’ weight was measured at baseline and at 3, 12, 24 and 60 months, and cholesterol HbA1c and blood pressure were measured at baseline and at 12 and 60 months. The decision problem was to determine whether the 12- and 52-week interventions were effective over the lifetime at reducing the incidence of disease and increasing QALYs for adults with a BMI ≥ 28 compared to a brief intervention and simulated no intervention scenario.

## Model comparison

## Models compared

The Health Checks model was chosen for comparison with the SPHR model. The model code was freely available allowing for an in depth understanding of the models, and the authors of the original model code agreed to support the model comparison throughout the process.

### School for Public Health diabetes prevention model

The School for Public Health Research (SPHR) diabetes prevention model is a microsimulation health economic model that describes individuals’ risk of type 2 diabetes, microvascular outcomes, cardiovascular disease (CVD), congestive heart failure, cancer, osteoarthritis, depression, dementia, and mortality in England. The model includes correlated trajectories of risk factors, including body mass index (BMI), systolic blood pressure, cholesterol and blood glucose, and annual changes in these risk factors impact the risk of health conditions in the model. Benefits are measured in QALYs, and the model uses a National Health Service (NHS)/personal social services perspective. The model was developed in the R software to examine the cost-effectiveness of type 2 diabetes prevention programmes; detailed information about the model can be found in a prior studies [17, 18] and in Table S1.

### Health Checks model

The HC microsimulation model was developed to examine the impact of the NHS Health Checks cardiovascular disease prevention programme in England on the risk of ischaemic heart disease, stroke, dementia, and lung cancer. The health checks intervention involved eligible simulated patients being invited for a health check, potentially followed by referral for one or more statin medications, antihypertensive medication, smoking cessation, and/or weight management depending on health status. This referral had the potential to impact the trajectories of risk factors in the model, including BMI, blood pressure, smoking status, and cholesterol. Benefits are measured in QALYs. No costs were included. Detailed information about the model, which was programmed in Python, can be found in a prior study [19] and in Table S1.

The models were compared qualitatively to identify structural differences (Table S1 of the supplementary material). Existing publications, technical descriptions and the model code were reviewed to document the model structures. Developers of both models contributed to the protocol and identified methodological challenges to the validation process.

### Standardising model setup

Models were standardised to ensure that model setup were the same across models. Supplementary material A and Table S3 provides a summary of the standardisation steps. In contrast to previous cross-model validations [6], we found that using a common set of parameter inputs was not feasible, as both models used multivariate risk equations that are conditional on multiple characteristics, and different methodologies for applying utility decrements.

The HC model was adapted in the following ways to reflect the model setup and baseline population characteristics used in the SPHR model.

- Baseline population was HSE 2014 (instead of 2012).
- Discount rate of 3.5% applied (instead of no discount).
- Baseline utility from HSE 2014 (instead of 1).

All standardisation steps were made to the HC model. Each standardisation step was conducted independently, and then all steps were applied simultaneously.

### Outcomes compared

To understand the differences between the models we conducted an in-depth assessment of consistency across multiple model outcomes, including uncertainty analyses and sub-group analyses. We examine include outcomes that will impact decision-makers (QALYs), we also examined aggregate outcomes, incremental values, intermediate outcomes which include diagnosis of health conditions and outcomes for relevant subgroups. Previous cross-model validation examples [6] have suggested that an acceptable magnitude of difference between model outcomes should be identified in advance of the model comparison. However, in the absence of cost estimates it was not possible to calculate the acceptable magnitude of differences based on decision impacts.

### Identifying aspects of model structure that explain differences in outcomes

We identified some adaptations that could be made to each model to make the model structures more similar. Because of the complexity of both models, it was impractical to change every aspect of one model incrementally to become like the other model and so we focussed on (a) the aspects of the model could be feasibly modified and (b) what we expected to make a difference to the outcomes of interest. Running iterations of the models in which, the model structures are made more similar can increase our understanding of how differences in model structure contribute to the differences in model outcomes. The options for model adaptations were pre-specified in the protocol. Of the pre-specified model adaptations specified in the protocol (Table S5 of the supplementary material) three model adaptations were selected in which the SPHR and/or the HC model were altered such that the models were more similar, and 4 scenarios were explored:

A. *Remove impact of treatment on HbA1c from SPHR.* The HC model was not designed to model the impact of changing HbA1c and so, the HBA1c trajectory did not impact on risk of diabetes or cardiovascular disease as is the case in the SPHR model. Therefore, the SPHR model was run without modelling the impact of the treatment on HbA1c trajectories to replicate the HC model structure.
B. *Same health conditions.* In addition to changes A, the models were adapted so that they had the same health conditions. Both models were run with only the conditions that the models had in common (diabetes, dementia, and CVD) removing microvascular complications, osteoarthritis and cancer from the SPHR model and lung cancer from the HC model. Other-cause mortality was not adjusted. Although this will result in slightly inflated life year estimates, adjusting mortality would have required a large model adjustment and the expected impact on mortality would be very small. Given the adjustment required and the small expected effect, we decided to compare model results without a full mortality adjustment.
C. *Use ELSA trajectories in the SPHR model*. The SPHR model was run with the trajectories of risk factors based on an analysis of the ELSA dataset. This differs from the base case in which trajectories from age 18 to 59 were based on analysis of the Whitehall II data set with trajectories for age 60 and over were based on ELSA. The modelling methods for using the ELSA data remained different.
D. *All structural changes applied.* Sensitivity analyses A, B and C were applied together to explore the impact of all adjustments combined.

## Results

### Impact of standardising the model setup

Three changes were made to the HC to reflect the model step-up used in the SPHR model.

- Baseline population was HSE 2014 (instead of 2012).
- Discount rate of 3.5% applied (instead of no discount).
- Baseline utility from HSE 2014 (instead of 1).

The results of each change to the model, then of all adaptations were applied simultaneously are shown in Table 1 alongside outcomes generated by the SPHR model. Discounting had the largest independent impact on absolute QALYs. All following model comparisons were conducted with all standardisation steps applied.

**Table 1:** Absolute and incremental QALYs generate by the SPHR and health checks model including standardisation steps

	Simulated Natural History	Brief intervention	12-week intervention	52-week intervention
*Absolute QALYs estimated by the SPHR Model (no standardisation steps applied)*				
Original model inputs	11.4 [10.5, 12.2]	11.4 [10.5, 12.1]	11.4 [10.5, 12.2]	11.4 [10.6, 12.2]
*Absolute QALYs estimated by the HC Model*				
Original model inputs	28.4 [27.7, 29.2]	28.4 [27.7, 29.2]	28.4 [27.7, 29.2]	28.38 [27.7, 29.2]
a.	28.3 [27.7, 29.0]	28.3 [27.8, 29.0]	28.3 [27.78, 29.0]	28.33 [27.8, 29.0]
b.	17.0 [16.7, 17.2]	17.0 [16.7, 17.2]	17.0 [16.7, 17.2]	17.01 [16.7, 17.2]
c.	21.6 [21.1, 22.0]	21.62 [21.2, 22.0]	21.62 [21.2, 22.0]	21.62 [21.2, 22.00]
a, b, and c applied	13.11 [12.8, 13.5]	13.12 [12.8, 13.5]	13.12 [12.8, 13.5]	13.12 [12.8, 13.5]
*Incremental QALYs estimated by the SPHR Model (no standardisation steps applied)*				
	0.0019 [-0.0499, 0.0416]		0.0248 [-0.0024, 0.0599]	0.0298 [-0.002, 0.0688]
*Incremental QALYs (vs. Natural history) estimated by the HC Model*				
Original model inputs	0.0305 [0.0153, 0.0438]		0.0293 [0.0171, 0.0439]	0.0308 [0.0171, 0.0434]
a.	0.0311 [0.0150, 0.0438]		0.0299 [0.0173, 0.0409]	0.0314 [0.0165, 0.0441]
b.	0.0122 [0.0090, 0.0169]		0.0113 [0.0080, 0.0145]	0.0125 [0.0076, 0.0170]
c.	0.0191 [0.0088, 0.0250]		0.0194 [0.0110, 0.0243]	0.0221 [0.0183, 0.0283]
a, b, and c applied	0.0078 [0039, 0.0125]		0.0079 [0043, 0.0126]	0.0085 [0048, 0.0129]

a. Baseline population was Health Survey for England 2014 (instead of 2012), b. Discount rate of 3.5% applied (instead of no discount), c. Baseline utility from HSE 2014 (instead of 1), QALY– Quality Adjusted Life Years

### Comparison of model outcomes

Table 2 shows the estimated QALYs and health conditions (per 1000) when no treatment effect is applied (for the original models, and for the HC model with standardisation steps applied). There were fewer strokes and CVD events and fewer cases of dementia in the HC model. The cases of dementia provided the largest difference in simulated health outcomes between the models. Between the original HC model and the standardised version, there are small differences in the number of events due to differences in the baseline population. The utility decrement for each condition is also shown in the table. The direct comparison of utility decrements is challenging because the HC model applies the decrement additively, whereas the SPHR model uses a multiplicative model. However, the health decrements in the SPHR model will have a greater impact on QALYs in an otherwise healthy population.

**Table 2:** QALYs and cases of and utility decrements associated with cardiovascular disease, dementia, and diabetes

		Health Checks model		Percentage difference	
	SPHR Model	Original	Standardisation steps applied	Original	Standardisation steps applied
QALYs	11.37 [10.51, 12.16]	28.4 [27.7, 29.2]	13.11 [12.76, 13.48]		15%
*Cases (per 1000)*					
CVD	411 [231, 632]	350 [345, 359]	350 [341, 360]	-15%	-15%
Stroke	208 [110, 324]	119 [114, 126]	119 [115, 127]	-42%	-42%
Dementia	229 [203, 287]g	118 [113, 125]	99 [96, 105]	-48	-57%
Diabetes	356 [164, 486]	561 [550, 570]	546 [535, 555]	+ 58%	+ 53%
*Utility Decrements*					
CVD	0.760	-0.12			
Stroke	0.629	-0.21			
Diabetes	0 (complications only)	0			
Dementia	0.478–0.93	-0.12			

*Note* the SPHR decrements are applied using the multiplication method; the HC uses the addition method. QALY– Quality Adjusted Life Year, SPHR– School for public health

*With standardisation steps applied

Table 3 shows the absolute and incremental QALYs for each simulated intervention group and natural history control. The total QALYs estimated by the HC model were higher in all treatment groups with no overlap in PSA credible intervals (Table 3). There was a large difference in incremental QALYs, and the credible intervals for the SPHR model overlapped the HC model. The SPHR simulated greater QALY gains for 12-week WW and 52-week WW relative to the Brief Intervention. Comparisons across socioeconomic groups found a stronger socioeconomic gradient in the SPHR model, and the results are reported in Table S6 in Supplementary Appendix B.

**Table 3:** Absolute QALYs and incremental QALYs versus simulated natural history in the SPHR and HC models

	Absolute QALYs		Incremental QALYs compared to simulated natural history		Incremental QALYs compared to simulated natural history (days)	
	SPHR Model	Health Checks Model*	SPHR model	Health Checks Model*	SPHR model	Health Checks Model*
Simulated natural history	11.3675 [10.5124, 12.1586]	13.1084 [12.7644, 13.4805]				
Brief intervention	11.3694 [10.5161, 12.1584]	13.1154 [12.7644, 13.4808]	0.0019 [-0.0499, 0.0416]	0.0078 [0.0039, 0.0125]	0.7 [-18.2, 4.6]	2.8 [1.4, 4.4]
12-week intervention	11.3923 [10.5479, 12.1737]	13.1157 [12.7647, 13.4809]	0.0248 [-0.0024, 0.0599]	0.0079 [0.0043, 0.0126]	9.1 [-0.9, 21.9]	2.9 [1.6, 4.6]
52-week intervention	11.3973 [10.5663, 12.1715]	13.1160 [12.7656, 13.4845]	0.0298 [-0.002, 0.0688]	0.0085 [0.0048, 0.0129]	10.9 [-0.1, 25.1]	3.1 [1.8, 4.7]

QALY– Quality Adjusted Life Year, SPHR– School for Public Health

*With standardisation steps applied

### Impact of iterative adjustments to identify drivers of differences in outcomes

#### Remove the impact of treatment on HbA1c from the SPHR

**Fig. 1:**
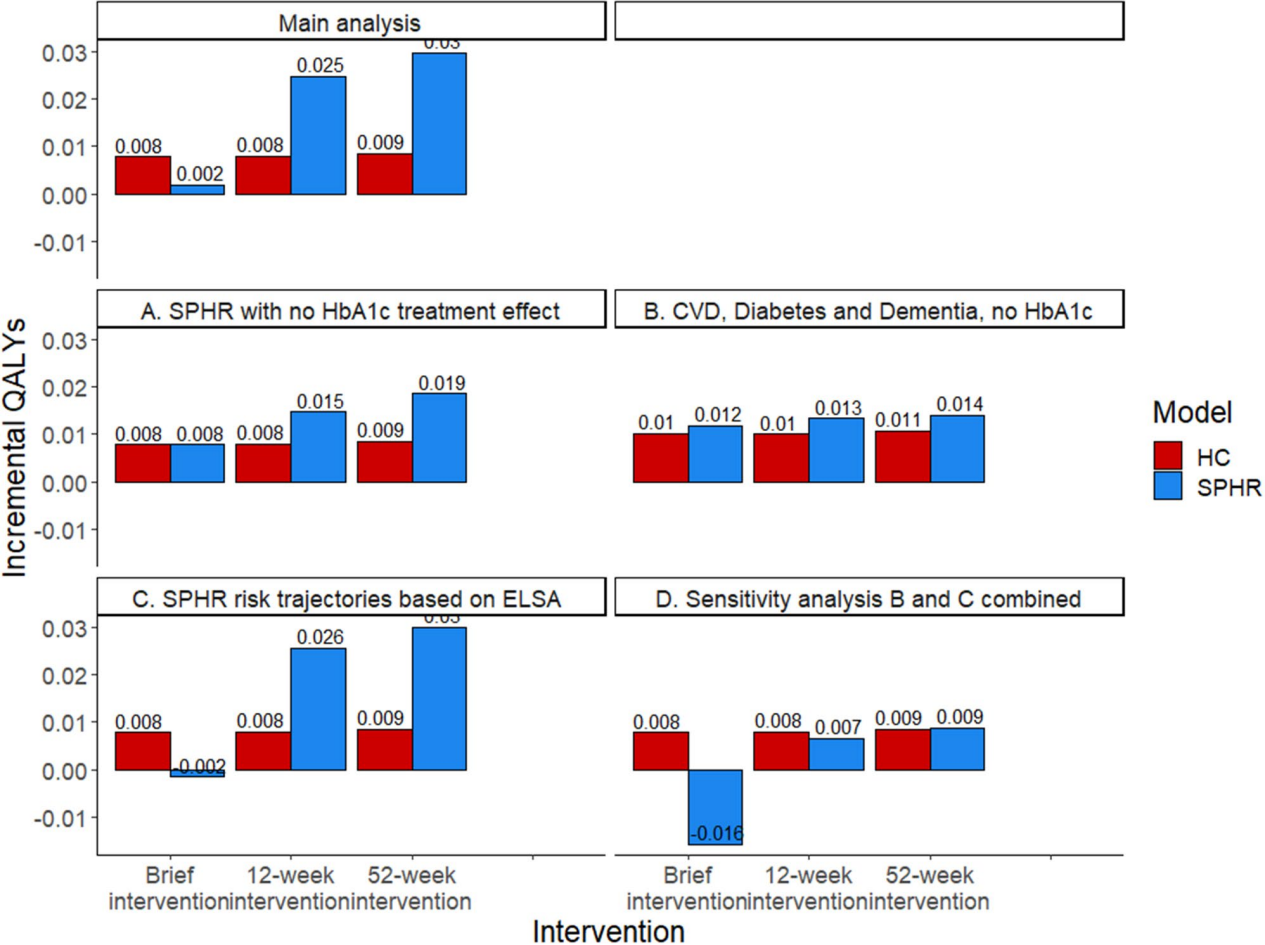
Incremental QALYs between each treatment scenario (brief intervention, 12-week intervention and 52-week intervention) and simulated no intervention scenario for each model (HC: Health Checks Model and SPHR: School for Public Health diabetes prevention model) for the main analysis and for four scenario where adjustments have been made to one or both models (**A**. impact of treatment on HbA1c removed from the SPHR model, **B**. Models run only with the condition represented in both models (diabetes, dementia, and CVD) plus scenario A, **C**. Estimate trajectories of risk factors based on ELSA dataset in the SPHR model, and **D**. Scenario B and C are combined so that all structural changes described were applied)

Making this adjustment resulted in more similar incremental QALYs between the two models, but the SPHR model still predicted a greater difference between the brief intervention and the 12-week and 52-week interventions than the HC model (Fig. 1, Table S7 of the supplementary material).

#### Same health conditions

The analysis was run on models adapted to include only the conditions that the two models have in common (diabetes, dementia, and CVD) removing microvascular complications, congestive heart failure, osteoarthritis and cancer from the SPHR model and lung cancer from the HC model. This was combined with the previous adaptation (i.e., the treatment effect on HbA1c was removed from the SPHR model). Absolute and incremental QALYs became more similar across models (Fig. 1, Table S7 of the supplementary material).

#### Use ELSA trajectories in the SPHR model

When the SPHR model was run with the trajectories of risk factors based on an analysis of the ELSA dataset rather than the Whitehall II dataset, there were very small changes to the incremental QALYs. There was a negative incremental QALY when comparing the brief intervention to natural history. This negative incremental QALY for the brief intervention was due to higher simulated BMI in the brief intervention arm compared with the simulated natural history using the ELSA trajectories. (Fig. 1, Table S7 of the supplementary material).

#### All structural changes applied

Sensitivity analyses A, B and C were applied together. As above, the brief intervention arm produces a higher BMI over time than the simulated natural history, which leads to a loss of QALYs in the brief intervention arm. (Fig. 1, Table S7 of the supplementary material).

Figure 1. Incremental QALYs compared to the simulated no intervention arm.

## Discussion

Estimates of long-term effectiveness of the weight management programme were similar using the SPHR and HC models after adjusting for explainable differences, namely, removing effects of weight management on HbA1c, limiting the models to a common set of health outcomes, and using a common dataset to derive metabolic trajectories. Since the SPHR model includes stronger evidence on the effect of weight management on HbA1c, the results of this cross-model validation support the existing cost-effectiveness analysis [4] and provide additional confidence in those estimates.

These findings reflect the suitbaility of the SPHR model for the deicision problem; the models original purpose, to evaluate diabetes prevetion strategies, resulted in a focus on diabetes-related risk factors (including HbA1c) and outcomes. There was a signifincant change in HbA1c in the intervention evaluated and the SPHR model enabled long-term modelling of this effect. In contrast, the HC model was developed to represent the impact of health checks for adults between the age of 40 and 74 and this health check could result in upto 4 treatments; statins, antihypertensives, smoking cession and weight management. This is reflected in the outcomes modelled (e.g., lung cancer) and diabetes was not a focus of the model. Therefore, one of the impacts (change in HbA1c) of the intervention could not be modelled in the HC model.

The process of cross-model validation resulted in a deeper understanding of the characteristics and sensitivities in the models. By identifying the features of the model that explain differences, it is possible to reflect on whether they should be included in the final analysis. The process of cross-model validation we conducted highlighted that the inclusion of HbA1c was the most important driver of differences in incremental QALYs; and that the inclusion of diabetes-related complications was most important for differences in total QALYs. These observations highlight the importance of modelling the interdependence between obesity and diabetes outcomes. The model comparison process can also inform future decisions around the development of both the existing models and new models. We conclude that cost-effectiveness analyses of weight management interventions should consider representing the intervention effects on HbA1c to avoid potentially underestimating the cost-effectiveness.

This analysis has several strengths. First, collaboration between model developers throughout the process enabled greater understanding of the models, enhanced error checking, discussion of results and setting priorities for comparison. This collaboration, along with access to all model code, facilitated a model comparison process where models can be incrementally adapted to isolate and understand drivers of differences in outcomes. Increase in the use of open source health economic models will support cross-model validations [20] as there will be more models for which full model code is available. Second, we have demonstrated how a process of cross-model validation can be used to compare complex public health microsimulation models. Existing validation methods and recommendations [6, 10] are not always practical for all model types. For example, the potential for differences in model boundaries, model structures, and specification of input parameters increases as the number of health outcomes increases, and public health models are more likely to adopt complex model structures [21]. This makes it difficult to draw conclusions about what is an acceptable difference between model outcomes, and there are likely to be a wide range of outcomes of interest, including prevalence of health conditions, costs and QALYs, and these may differ between models. Finally, the in-depth comparison can be used to inform future model development. Squires et al. recommend that when developing and justifying the structure of a new model, any existing health economic models should be reviewed [22]. Cross-model validation has the potential to provide information for this stage of development, but it is often reported very briefly as part of model development if at all, and there is a missed opportunity for this to inform future model development. A greater emphasis on reporting cross-model validation in detail, either as a separate publication or within supplementary material, can enable the results to have an impact beyond the decision problem. Our method can be a starting point that can be used and tested in other model comparisons.

While conducting the model comparison, we identified challenges that are likely to be common to cross-model validation, so we discuss the justification for the approach taken. First, each model was developed for a different purpose and then adapted to examine the long-term effectiveness of the weight management intervention. They were therefore structurally different, which made it challenging to decide on the extent to which the models can be standardised, and which changes to make when conducting iterative adjustment to the model. Broadly, in standardisation we intended to ensure that the model setup is consistent in terms of the eligible population, time horizon, discount rate, and perspective. This deviates from other cross-model validation approaches that aim to align model inputs prior to examining structural differences [6]. We found that using a common set of parameter inputs was not feasible, particularly for multivariate risk equations that are conditional on multiple characteristics and different methodologies for applying utility decrements. These differences are more likely to arise in microsimulation models, which allow for complex relationships to be represented. We then intended to interrogate the structural assumptions and data sources informing long-term health outcomes. Although there were multiple potential iterations, we could have conducted, resource constraints meant that our choice of which changes to make was informed by the outcomes of the standardisation steps, the initial model comparison, and discussions with the model developers.

Second, it was difficult to decide which of the multiple potential outcomes to examine and report. Public health economic evaluations often need to consider a broad range of outcomes, time horizons, and cost perspectives [22]. As a consequence, we decided that the validation process should aim to make comparisons across outcomes that will inform the validation process or impact decision-making. In public health microsimulation models, this may include subgroup analyses, as these results may impact conclusions regarding equity impacts for an intervention. Detailed cross-model validation that provides an in-depth assessment of consistency across multiple model outcomes, including uncertainty analyses and subgroup analyses, has the potential to provide a much greater understanding of the differences between the models, as demonstrated. While the validation should include outcomes that will impact decision-makers (costs and QALYs), it should also extend to include other intermediate outcomes to help understand what may be driving the differences. However, steps taken to understand drivers of difference may involve changing isolated factors of the model and caution should be taken when interpreting these results. For example when we examined the impact of including the same conditions only by removing health conditions from each model, we made the decision not to adjust other-cause mortality due to the extent of structural changes required. These results inform our understanding of the drivers of differences between the models but the outcomes of this scenario could not be used to inform decision-making. Caution should be taken when making changes to individual parts of the model to consider the unintended consequences on other outcomes. Finally, previous cross-model validation examples [6] have suggested that an acceptable magnitude of difference between model outcomes should be identified in advance of the model comparison. However, in the absence of cost estimates, it was not possible to calculate the acceptable magnitude of differences based on decision impacts.

While conducting the cross-model validation, we developed some proposed recommendations (detailed in Supplementary Material, Appendix D). However, the current cross-model validation is based on a comparison of two broadly similar microsimulation models, which provides a useful but limited overview of opportunities and challenges for model comparison. A different set of challenges may arise if validating a detailed microsimulation model against a different modelling structure, for example, a multistate lifetable model structure often used in public health evaluations. It is possible that future cross-validation will build on this, and other existing detailed model comparisons, to help to develop and refine a set of recommendations that can be formalised into a standardised best practice guide for modellers.

## Conclusions

The cross-model validation process supported the findings of the initial cost-effectiveness analysis of this weight management programme and identified which structural differences contributed most to differences in outcomes. The process we have reported addresses the need for more examples of cross-validation approaches for public health economic models and is a preliminary step in the development of cross-model validation guidelines specific to complex interventions.

### Electronic supplementary material

Below is the link to the electronic supplementary material.

Supplementary material: Supplementary Appendix A: Published guidance on cross-model validation. Supplementary Appendix B: Protocol to compare and cross-validate models. Supplementary Appendix C: Summary report of cross-model Validation. Supplementary Appendix D: Proposed recommendation for Cross-model validation

## Data Availability

No new datasets were generated or analysed during the current study. The WRAP trial data are not publicly available. Participant consent allows for data to be shared for future analyses with appropriate ethical approval. Non-identifiable data and analysis code can be made available to bona-fide researchers on submission of a reasonable request to datasharing@mrc-epid.cam.ac.uk. The principles and processes for accessing and sharing data are outlined on the UK Medical Research Council Epidemiology Unit Data Sharing Portal: epi-meta.mrc-epid.cam.ac.uk. Meta data for the WRAP study is available at https://epidata-ext.mrc-epid.cam.ac.uk/ddic/overview/WRAP/. The source code for the Health Checks model is available under licence from a GitHub repository (https://github.com/chjackson/healthchecks).

## Abbreviations

SPHR: School for Public Health Research
HC: Health Checks
WW: Weight watchers
ISPOR-SMDM: International Society for Pharmacoeconomics and Outcomes Research–Society for Medical Decision Making
QALY: Quality-Adjusted Life Year
WRAP: The extended and standard duration Weight-loss programme Referrals for Adults in Primary care
CVD: Cardiovascular disease
BMI: Body Mass Index
NHS: National Health Service
HSE: Health Survey for England
