# Supplementary material for: Cross-model validation of public health microsimulation models; comparing two models on estimated effects of a weight management intervention
Source: BMC Public Health. 2024 Mar 12;24:764. doi: 10.1186/s12889-024-18134-4 (PMC10935815; doi:10.1186/s12889-024-18134-4)
Supplement: Supplementary file 1 — Supplementary material: Supplementary Appendix A: Published guidance on cross-model validation. Supplementary Appendix B: Protocol to compare and cross-validate models. Supplementary Appendix C: Summary report of cross-model Validation. Supplementary Appendix D: Proposed recommendation for Cross-model validation [file 12889_2024_18134_MOESM1_ESM.docx]

# Supplementary Material

Supplementary Appendix A: Published guidance on cross-model validation

Table S1: Summary of published guidance on cross-model validation

| Source of guidance | Context of guidance | Motivation for guidance | Specific instructions |
| --- | --- | --- | --- |
| ISPOR-SMDM Modeling Good Research Practices | Recommendations for transparency and validation developed by an International Society for Pharmacoeconomics and Outcomes Research (ISPOR) and the Society for Medical Decision Making (SMDM) task force | To increase confidence in models results and gain understanding of the impacts of methods on outcomes. | - Model results should be compared to analyses of the same or similar decision problems to gain insights from similarities and differences in results |
| AdViSHE | Checklist tool for model developers to complete during/after model development developed by 47 experts in health economic modelling and decision making | To increase confidence in decision models, standardise and record validation processes and reduce workload of researchers. | Steps on checklist:   - Have the model outcomes been compared to the outcomes of other models that address similar problems? If yes, please provide information on the following aspects: - Are these comparisons based on published outcomes only, or did you have access to the alternative model? - Can the differences in outcomes between your model and other models be explained? - Please indicate where this comparison is reported, including a discussion of the comparability with your model.   If no, please indicate why not. |
| Lessons Learned from a Cross-Model Validation | Lessons learned from a single cross-model validation of two breast cancer models | To perform a cross-model validation and suggest guidance based on this experience | - Compare model framework including the target population, intervention, time frame and health outcomes qualitatively to identify differences. - Run models using the same input data. - Start with a comparison on natural history parameters. - Compare undiscounted point estimates, then discounted point estimates, and then cost-effectiveness results. - Compare both absolute and relative differences and confidence intervals across models. - If outcomes are different, examine the impact of model structure. |

Supplementary Appendix B: Protocol to compare and cross-validate Model 1: School for Public Health Research diabetes prevention model (SPHR) with Model 2 Health Check model (HC)

This exemplar protocol has been designed based on the original model comparison plan and the learning from the case study implementation process.

**Background**

To encourage greater confidence in the cost-effectiveness findings, we will undertake comparative modelling of the WRAP-UP trial to consider the impacts of structural assumptions. This will involve comparing results from the SPHR Diabetes Prevention model (Breeze et al., 2017) and the NHS Health Checks model from Cambridge (Mytton et al., 2018). The aims of the model comparison were to assess whether the models generate similar aggregate health economic outcomes, which would imply similar decision recommendations across model. A secondary aim was to understand what model structures are important in explaining any differences in model outcomes.

**Simulation models to be compared**

The SPHR model is a microsimulation health economic model that describes individuals’ risk of type 2 diabetes, microvascular outcomes, cardiovascular disease (CVD), congestive heart failure, cancer, osteoarthritis, depression, dementia, and mortality in England. Benefits are measured in QALYs and the model uses an NHS/personal social services perspective.

*Health Checks (HC) model.* The HC model is a microsimulation model developed to examine the impact of the NHS Health Checks cardiovascular disease prevention programme in England on the risk of ischaemic heart disease, stroke, dementia, and lung cancer. The health checks intervention involved eligible simulated patients being invited for a health check, potentially followed by referral for one or more of statin medication, antihypertensive medication, smoking cessation, and/or weight management depending on health status. Benefits are measured in QALYs. No costs were included.

**Access to model code**

The Health Checks model code is available on GitHub (https://github.com/chjackson/healthchecks) and the model developed will provide data files, in their processed format so that they can be used directly in the model. This includes the HSE baseline population files, the matched biomarkers ELSA trajectories, the life table data for disease-specific and all-cause mortality and anything else necessary to run the model.

Developers of the Health Checks model will provide any information about how the data was processed to aid to understanding. Researchers involved in the development of both models met for ongoing trouble-shooting support throughout the model comparison process, when necessary. Researchers conducting the model validation have existing access and knowledge of the SPHR model.

**Differences between Models**

The differences between the SPHR and HC models are outlined in Table S2.

Table S2: Difference between the School for Public Health diabetes prevention model and the Health Checks model.

| **Element** | **SPHR Model** | **HC Model** |
| --- | --- | --- |
| Model Code | Written in R. Parameters exported from Excel to data file imported into model. | Written in Python. Parameters written directly into a parameters script. |
| Model Structure | Microsimulation with annual cycles and lifetime horizon. NHS /social services perspective. Discount rate of 3.5% costs and QALYs | Microsimulation with annual cycles and lifetime horizon. No costs are included. No discount rate. |
| Baseline Population | Health Survey for England ^1^ (HSE) 2014. All individuals over 16 were included, with multiple imputation methods used to combat missing data. Use of survey weights to ensure population resembles England. Subsetting of population to ensure trial eligibility. | HSE 2009-2012. The modelling conducted previously only included individuals aged 40-45. Only individuals with recorded cholesterol, SBP, DBP and HbA1c are included. Synthetic population drawn using stratified sampling with replacement to ensure England 2011 census sex/ethnic balance. |
| Baseline prevalence | Baseline prevalence of conditions was determined by conditions reported in the HSE sample. Family history of diabetes was imputed based on HbA1c and ethnic origin (using Whitehall II ^2^ data). Individuals with a history of cardiovascular disease (CVD) were assigned to a health status of either stable angina, unstable angina, myocardial infarction, or stroke based on responses to HSE. Individuals reporting stroke were assigned to stroke, heart attack/angina to unstable angina and MI at random using distributions estimated in a previous HTA. | A proportion of the population were assumed to have CVD, according to age- and sex-specific prevalence of IHD and stroke from 2012 HSE data, adjusted for consistency with concurrent mortality data using the DISMOD program Individuals with highest QRISK2 score at baseline were then assigned to have either ischemic heart disease (IHD) or stroke. It’s assumed that nobody had dementia or lung cancer at baseline. |
| Annual incidence of disease | **GP attendance** was based on age, sex, BMI, ethnicity, and health outcomes. Assumed that individuals eligible for anti-hypertensive treatment or statins will be identified through opportunistic screening if they meet certain criteria (history of CVD, major microvascular complication, diabetes, IGR, high SBP) and attend the GP for at least one visit in the simulation period.  Assumed that there are three, non-mutually exclusive outcomes from the vascular checks or opportunistic screening. Firstly, that the patient receives **statins** to reduce cardiovascular risk (QRISK greater than 20%). Secondly, that the patient has high blood pressure and should be treated with anti-**hypertensive** medication (SBP above 160, or SBP over 140 and history of CVD, diabetes, or a CVD risk >20%). **Diabetes** diagnosis is based on the QDiabetes 2018 risk algorithm and the HbA1c. An individual is diagnosed with Diabetes if they are assessed to experience a diabetes diagnosis in the next 10 years using the QDiabetes algorithm and have two HbA1c tests scores above 6.5 in that period.  The probability of the first **CVD** event is estimated from the QRISK2. The 1-year estimates were estimated through calibration to match the CVD incidence in the WRAP trial. Type of event was based on a previous HTA analysis. The probability of following events was based on the same analysis. **Congestive heart failure** was included as a separate cardiovascular event and was conditional on age, diabetes diagnosis, BMI and SBP.  Assumed that **microvascular complications,** including **renal failure, amputation, foot ulcer, and blindness,** only occur in individuals with HbA1c>48 mmol/mol (6.5%) using both versions of the UKPDS outcomes model.  Incidence rates for **breast and colorectal cancer** was based on the EPIC study, with an adjustment for BMI. **Osteoarthritis** was conditional on diabetes and BMI based on an Italian study. Individual can develop **depression** in any cycle of the model assuming that a diagnosis of diabetes and/or CVD increased the incidence of depression. **Dementia** is based on the THIN dementia score and is conditional of age, gender, deprivation, BMI, smoking status, diabetes, depression, stoke atrial fibrillation. | **Hypertension** diagnoses were simulated based on the simulated blood pressure data. A random 5% of people with systolic blood pressure of over 150mmHg were assumed to be diagnosed (and treated) with hypertension each year.  **Diabetes** diagnoses were simulated based on diabetes diagnosis data in ELSA. Diabetes was also assumed to be diagnosed in 5% of individuals per year whose HbA1c level is above 6.5. Once it is predicted from ELSA that a person would be diagnosed with diabetes within a four-year timeframe, the year of the diagnosis was chosen at random.  It’s assumed that the background trajectories, representing a ‘real-world’ population will account for people being given risk factor-modifying treatments outside the NHS Health Check programme.  Annual incidence of **CVD** was computed using a combination of QRISK2 and inferences from prevalence (HSE) and mortality data based on the DISMOD program. The specific event, either IHD or stroke, was based on the relative incidence of each disease.  The same method was used to calculate the annual probability of **dementia** onset. The CAIDE score estimates the 20-year risk of dementia, which was converted to a 1-year risk based on estimates of the average incidence in the population (by age and sex) estimated from routine data using DISMOD. For simulated individuals aged 60 years or older, it was assumed that each simulated individual stayed on the same risk percentile they were at when aged 60 years. A simulated individual’s increase in absolute risk with age is informed by the estimates based on DISMOD.  **Lung cancer** incidence was also based on national cancer registry data, adjusted for consistency with mortality data using DISMOD. There were separate estimates for smokers and non-smokers based on the proportion of incident cases of lung cancer attributable to smoking. Only current smokers are counted as smokers. Ex-smokers are considered as non-smokers.  Once an individual in the model has one of these diseases, it is assumed that they had it for the rest of their life. This was associated with a higher mortality and lower health-related quality of life. |
| Risk Factor Trajectories | Trajectories for BMI, HbA1c (glycaemia), SBP, total cholesterol and HDL cholesterol based on analysis of the Whitehall II dataset^3^ (age <65) and ELSA (age>=65) data. Latent growth curve modelling of longitudinal data produced parameters enabling simultaneous estimation of all 5 risk factors for the next modelled year taking into account correlations and variation between individuals. | Trajectories for BMI, HbA1c, SBP, DBP, LDL cholesterol and HDL cholesterol were based on ELSA data. In each modelled year and for each metabolic factor independently, individuals are matched to an individual within ELSA within the same category of risk predictor variables. Change in risk is based on next ELSA observation (4 years later) with linear adjustment for time. |
| Included Conditions/ Events | Diagnosis and treatment of hypertension, high CVD risk and diabetes. CVD events through QRISK2, secondary CVD events through a previous statins HTA, diabetes complications through UKPDS, congestive heart failure through Framingham, dementia through THIN & MMSE score, breast & bowel cancer, osteoarthritis, and depression. No lung cancer modelled. CVD mortality (at point of event only), cancer and dementia mortality (lifelong following diagnosis) and other cause mortality. | Diagnosis and treatment of hypertension, high CVD risk, overweight & smoking. Diagnosis of diabetes (no treatment) is based on matched person in ELSA data set. CVD events using QRISK2 and DISMOD, no secondary CVD events modelled, dementia using CAIDE, lung cancer using DISMOD. No diabetes complications, congestive heart failure, breast or bowel cancer, osteoarthritis or depression modelled. |
| Mortality | Cardiovascular mortality is included as an event within the QRISK2 and the probability of subsequent cardiovascular events was obtained from an HTA assessing statins.  Cancer mortality rates were obtained from the Office of National statistics. The age-adjusted 5-year survival rate for breast cancer and colorectal cancer were used to estimate an annual risk of mortality (3.37% and 11.16% for breast and colorectal respectively) assuming a constant rate of mortality. Mortality rate does not increase due to cancer beyond 5 years after cancer diagnosis.  All-cause mortality (risk of death except CVD and cancer) rates by age and sex were extracted from the 2014 ONS life tables subtracting the number of deaths related to cardiovascular disease, diabetes, dementia, breast, and colorectal cancer from the all-cause mortality total to estimate other cause mortality rates by age and sex. An increased risk of mortality was assigned to individuals with diabetes using data from a published meta-analysis. All-cause mortality was inflated after diagnosis of dementia. | CVD mortality, lung cancer mortality, diabetes mortality and dementia mortality and other cause mortality were estimated. Annual age, sex and disease-dependent case fatality rates by age were estimated from routine data sources using the DISMOD II v1.05 program.  It was assumed that some people who had a CVD event died at the time of that event; it is assumed that there was a probability of 0.5 that a CVD event was a myocardial infarction or a stroke and that there was an instant fatality risk of 0.3 for both a myocardial infarction and a stroke. The probability of death from obtained from DISMOD was adjusted so that it represents only non-event mortality for survivors of these events or people with CVD who have not experienced them yet.  Other-cause mortality was deduced based on (age-sex-dependent) data on all-cause mortality, cause-specific mortality, and prevalence from DISMOD. |
| Smoking | Baseline risk factor but no quitting is simulated. | Smoking included as risk factor and longitudinal changes in smoking status is based on ELSA. Simulated changes in smoking status were adjusted by reducing the 10-year quit rate from the average 6.5% observed in ELSA to 5%, and by increasing the 2-year relapse rate to 37%. Quitting was defined as a change from any of the smoking categories to not smoking, and relapse as a change from being an ex-smoker to a moderate smoker. Individuals were defined as ex-smokers if there are two consecutive records in ELSA of not smoking. |
| Interventions | Model has been used to analyse a range of interventions. Can include any intervention impacting on one or more of BMI, HbA1c, SBP or cholesterol, acting at model start. Intervention effects can be specified for each year post-intervention and/or undergo linear reduction/regain until specified duration of effect expires. The effect can be implemented by altering the natural history trajectory by a certain value.  Eligibility for an intervention and the attendance or uptake can be added in.  Interventions that impact on BMI can indirectly impact on the other metabolic risk factors via the change in BMI. | The model has been designed to examine health checks for people aged 40 to 74 who had not yet been diagnosed with CVD, diabetes, or hypertension. Individuals could still be diagnosed with hypertension or diabetes outside of the health check (5% of those with a high BP and 5% of people with high HbA1c, not diagnosed in ELSA).  On average individuals will be invited for a health check once every 5 years. Attendance is dependent on gender, age, ethnicity deprivation, smoking status and QRISK2 score. Assumed 5% of ineligible individual attended for a health check. At the simulated health check visit, simulated individuals were assumed to be offered one or more of four treatments: a statin, hypertensive medication, smoking cessation, or weight management.  2.05% or 14.23% of individuals are prescribed statins for those with a QRISK score of under or over 20%. Taking statins results in lower total cholesterol and high HDL levels (gender dependent) and reduction in the QRISK2 score based on calibration such that reduction in CVD events aligns with trial data. It was assumed that statin adherence was 50% and that 5% stop taking medication after a year (return to background trajectory).  1.54% or 2.48% of individuals are prescribed anti-hypertensives for those with a QRSIK score of under or over 20% (if SBP over 140). Anti-hypertensive medication resulted in lower systolic and diastolic blood pressure (age and gender dependent). It was assumed that antihypertensive adherence is 55% adherence and 5% stop taking medication after a year (return to background trajectory)  A proportion (14.6%) of those referred to smoking cessation (3.6%) were assumed to have quit after one year. 27.5% of eligible individual are referred to weight management. Everyone attending at least one session of weight management (assumed to be 50% of referred) lost weight by a year, resulting in lowered BMI assuming gradual linear weight regain over 5 years. For individuals treated with statins, anti-hypertensive medication or weight management via a health check, a treated trajectory is computed by shifting the background (no health check) trajectory during the period on treatment.  Health check can be altered to include only certain changes (e.g., weight change only). Treatment effects are assumed to be the same for everybody receiving the treatment. |
| Utilities | Utility decrements for CVD, dementia, diabetes complications (not diabetes alone), congestive heart failure, cancers, osteoarthritis, and depression applied to age adjusted baseline utility are combined multiplicatively. Some decrements are transient, others lifelong. There are decrements for age (and BMI in some model versions). | Based on EuroQol, utility decrements for heart disease, stroke, dementia, and lung cancer, are lifelong once diagnosed. Separate decrements given for any 2, 3 or 4 conditions. Decrements for age and socioeconomic deprivation. |
| Costs | Unit NHS and PSS costs for all included conditions/events in the model. Also, possible to include intervention costs. | No costs modelled. |
| Outcomes | Life years and QALYs, CVD events, diabetes diagnoses, costs. Outcomes from a range of different subgroups. | Life years and QALYs, CVD events, premature deaths prevented (<80), disease free at age 80. Outcomes by IMD quintile. |

**Base case model validation**

*Baseline population.* Both the models used Health Survey for England (HSE) ^15^ data however the HC model used a combination of all the data collected between 2009 and 2012. This data was restricted to individuals for which there was data on all required input variables (diabetes diagnosis, hypertension diagnosis, sex, age, index of multiple deprivation (IMD), systolic blood pressure, treatment for hypertension, family history of CVD, smoking status, Townsend score) and to individuals aged 30 to 74. The SPHR model used HSE data from 2014, used all the adult individuals in this wave of the dataset (depending on eligibility of specific intervention) and used multiple imputation to replace missing values of the required variables. Therefore, although both models used HSE data, the different waves of data used has the potential to introduce differences into the model. Therefore, to standardise the input to be the same in both models, the imputed 2014 data used for the SPHR was used in both models.

*Baseline utility.* In the original HC model, it was assumed that all individuals in the baseline population started with a utility of 1. The utility collected in the baseline HSE data was used to match the SPHR model.

*Intervention effect*. The HC model implements the effect of a weight loss intervention by assuming the same weight change for each person in the model. This weight change is applied to the background trajectory of BMI, which is estimated, along with the trajectories of other disease risk factors, using matching to observed longitudinal data (English longitudinal study of ageing.; ELSA ^16^). In contrast, the SPHR model estimates the intervention effect on BMI based on several factors including age, gender, and previous BMI changes. The HC model was altered such that the intervention effect on BMI was conditional on the same factors as used in the SPHR model. Both the SPHR and HC models modelled trajectories for BMI, systolic blood pressure, total cholesterol, HDL cholesterol and HbA1c. Whereas, only in the SPHR model did intervention effects on HbA1c impact long-term health outcomes.

*Discounting.* The HC model does not include a discount rate for QALYs and so discounting was added to match the SPHR model (discount rate of 3.5%).

*Time Horizon.* Lifetime horizon was implemented for all comparisons.

Table S3: Base case model specification to enable standardisation of models

| Baseline population | Population eligible to receive WW. The population will be randomly sampled from the HSE 2014. |
| --- | --- |
| Time horizon | Lifetime |
| Discount rate | 3.5% |
| Cost perspective | No costs included in the analysis. |
| Interventions | Brief intervention  12 week WW  52 week WW |
| Treatment effect | Differences in BMI (1 year, 2 years and 5 years), HbA1c (1 year and 5 years). |

Changes to SPHR model required for base case analysis

The SPHR model will already be adapted to model the WRAP-UP trial by the time model comparison takes place, so only a few changes will be required to enable model comparison to take place:

- We will extend the number of outcomes possible from the SPHR model to include all of those modelled as default in the HC model. This will include premature deaths prevented and numbers disease free by age 80

Changes to HC Model required for base case analysis

The HC model will require considerably more adaptation to enable it to model the WRAP-UP trial:

- The population will be modified to model those eligible for the WRAP-UP intervention. This will mean selecting a multi-aged population fulfilling the study criteria (rather than all people aged 40-45) and altering the stratified sampling to obtain a population with the age, gender, comorbidity, and socioeconomic profile of the eligible population.
- Our base case proposal is to use the health check intervention functionality to represent the WRAP-UP intervention, but the only consequence of this would be weight management (i.e., no statin or antihypertensive uptake, or smoking cessation).
- The weight management intervention will be altered to incorporate changes in trajectories other than just the BMI trajectory (i.e., HbA1c, SBP and cholesterol), and to enable 5 years’ worth of trajectory alterations to be inputted.
- Opportunistic uptake of statins and antihypertensives would be maintained in the model intervention and comparator arms, but opportunistic uptake of smoking cessation as this is not affected by the intervention.
- The HC model does not include NHS costs. We do not propose to add these in due to as conducting this complex adaptation would not be the scope of the model validation in which the focus is to compare existing model structures.
- We will add functionality to present model outcomes by population subgroups that are not already included in the HC model but are included in the SPHR model. This will include stratification of outcomes by diabetes diagnosis at baseline.
- Baseline utility values were replaced with data from the Health Survey for England population. The default setting in the model assigned the population with a utility of 1 at baseline.

**Model outcomes and sub-groups for comparison**

***Outcomes***

Model outcomes were lifetime Quality Adjusted Life Years (QALYs) and a number of health conditions. The models differ in the health conditions that are simulated and so comparisons were made only for conditions that are outcomes in both models (all CVD, stroke, dementia, and diabetes). The model outcomes were compared on natural history estimates (no intervention) and when intervention effects (based on the WRAP study^17^) are applied. Outcomes for subgroups based on IMD quintile were examined. QALYs were used as the primary outcome, as this represents a composite measure of length and quality of life and is measured in both models. The HC model didn’t estimate costs these were not included as outcomes. (See Table S4 of the supplementary material)

Table S4: Summary of model outcomes to compare in base case analysis

| Disease specific Outcomes | Cardiovascular disease, stroke, dementia, diabetes |
| --- | --- |
| Aggregated outcomes | Total QALYs, Life Years. |
| Sub-group analyses | IMD quintile, diabetes diagnosis. |

**Comparative Analyses to Test Structural Uncertainty**

The possible analyses and associated adaptations to the SPHR and HC models are in Table S5.

Table S5. Additional scenario analyses to modify structural and parameter differences between models.

| **Scenario** | **Description** |
| --- | --- |
| Sc1. Trajectories Comparison | The SPHR model could be adapted to include or approximate the trajectories structure from the HC model, with all other components of the model staying the same. Two options for how this might be done include:   1. Apply an adjustment factor to the SPHR trajectory model to make it match with summary data e.g., adjust the average BMI long term trajectory slope. 2. Programme the HC model structure into the SPHR model |
| Sc2. CVD Risk  Comparison | The SPHR model could be adapted to model CVD risk in the same way as the HC model, with all other components of the model staying the same. This would involve writing the DISMOD risk equation into the SPHR model, removing secondary CVD events from the SPHR model and restructuring QALYs appropriately. |
| Sc3. Diabetes | The HC model currently includes HbA1c and opportunistic diagnosis of diabetes through ELSA and annually in 5% of people with HbA1c >= 6.5%. This differs from the SPHR model, which diagnoses diabetes opportunistically in people who visit the GP with HbA1c above a personal threshold. Align the models by removing the impact of changes to HbA1c on diabetes diagnosis in the SPHR model. |
| Sc4. Other Conditions Comparison | Once diagnosed, diabetes in the HC model acts on CVD risk and mortality risk, whereas in the SPHR model diabetes acts on risks of CVD plus other conditions including microvascular disease, but does not impact directly on mortality. Microvascular disease is not included in the HC model and neither are a range of other conditions included in the SPHR model (breast and bowel cancer, osteoarthritis, depression, and congestive heart failure), but the HC model does include lung cancer, which the SPHR model does not.  A set of analyses could be done to look at the impact of modelling diabetes using HC model methods and including additional conditions as follows:   1. Align SPHR model with HC model in terms of opportunistic diagnosis of diabetes and impact of diabetes on CVD risk and mortality. 2. Remove microvascular complications from the SPHR model. 3. Remove breast and bowel cancer, osteoarthritis, depression, and congestive heart failure from SPHR model, plus remove lung cancer from HC model. 4. Combination of above scenarios. |
| Sc5. Opportunistic Diagnosis and Treatment Comparison | The HC and SPHR models both include opportunistic diagnosis and treatment of eligible people with anti-hypertensives and statins, but pathways and parameters for these differ between the two models. The SPHR model could be adapted to model opportunistic diagnosis and treatment of people with hypertension/high CVD risk in the same way as the HC model. |
| Sc6. Dementia Risk Comparison | The HC and SPHR models include different risk scores for modelling dementia. A set of analyses could be done to look at the impact of modelling dementia using HC model methods as follows:   1. Remove the progression of dementia functionality from the SPHR model, as this is not present in the HC model. 2. Change the risk function for incidence of dementia in the SPHR model to be that used in the HC model. 3. Combination of above scenarios. |
| Sc7.Utilities  Comparison | The SPHR model could be adapted to model utilities in the same way as the HC model. |

Supplementary Appendix C: Summary report of cross-model Validation School for Public Health Research diabetes prevention model (SPHR) with Model 2 Health Check model (HC)

**Base case model validation**

Health Outcomes

There were differences in the number of health conditions diagnosed in the models. There was a larger number of simulated individuals diagnosed with diabetes in the HC model compared to the SPHR model. This may be because the simulated individuals were matched with the individuals in the ELSA dataset and the rate of diagnoses here may be higher as cases of diabetes are identified as part of the study which may not have been identified ordinarily^15^. Furthermore, the health check model included diabetes only as a risk factor for CVD rather than a condition in itself, therefore there was no utility decrement associated with this diagnosis, and so despite the higher cases, it has less impact on the QALY outcomes than would be the case in the SPHR model. The number of CVD cases was similar across models although a higher proportion of these were strokes in the SPHR than for the HC model in which it was assumed that the remainder were IHD. This highlights the impact of the methods and data sources on the outcomes. It’s not possible to be sure of which is a more accurate estimate but being aware of how these methods can result in higher or lower cases of conditions than alternative methods can inform interpretation of outcomes. This is particularly important if the outcomes are close to a decision-making threshold, for example, close to the willingness-to-pay threshold when estimating cost-effectiveness and if using an alternative method might result in a different decision.

Aggregate QALYs

The comparison of QALYs in the base case analysis indicates that the SPHR model simulates lower QALYs than the HC model. However, the incremental QALY reported for the brief intervention, WW 21 weeks and WW52 weeks were larger for the SPHR model (Table 4 main manuscript).

Sub-group analyses by socioeconomic groups

For both models the lowest quintile (least deprived) was associated with the highest total QALYs and the highest quintile (most deprived) was associated with the lowest number of QALYs (Table S6). When comparing the brief intervention and 12-week intervention to the natural history, the intervention was most effective for most deprived quintile for both models. The HC model estimated that the 52-week intervention would be most effective for IMD quintile 2, whereas the SPHR estimated the intervention to be most effective for the IMD quintile 5. The impact of the intervention of the subgroups by IMD were broadly very similar. The differences observed that the 52-week intervention was most effective for IMD quintile 2 (HC model) compared to IMD quintile 5 (SPHR model), may be because the HC model assumed that deprivation impacted on incidence but not directly on mortality. Despite differences, both models estimated the interventions were generally more effective for more deprived subpopulations.

Table S6. Absolute and incremental QALYs versus do nothing scenario for different deprivation quintiles

|  | SPHR | | | | | HC | | | |  |
| --- | --- | --- | --- | --- | --- | --- | --- | --- | --- | --- |
|  | Do Nothing | Incremental QALYs | Do Nothing | Incremental QALYs |  |  |  |  |  |  |
|  |  | Brief Intervention | 12-wk WW | 52-wk WW |  | | Brief Intervention | 12-wk WW | 52-wk WW | |
| IMD 1 (least deprived) | 12.3641 | -0.0062 | 0.0181 | 0.0225 | 13.8831 | | 0.0056 | 0.0052 | 0.0062 | |
| IMD 2 | 11.3359 | 0.0001 | 0.0241 | 0.0293 | 13.2723 | | 0.0094 | 0.0094 | 0.0112 | |
| IMD 3 | 11.0883 | 0.0015 | 0.0245 | 0.0298 | 12.7038 | | 0.0086 | 0.0083 | 0.0088 | |
| IMD 4 | 11.1816 | 0.0050 | 0.0273 | 0.0323 | 13.0627 | | 0.0064 | 0.0074 | 0.0080 | |
| IMD 5 (most deprived) | 10.9483 | 0.0079 | 0.0291 | 0.0344 | 12.6398 | | 0.0099 | 0.0094 | 0.0092 | |

Model Adaptations Implemented

Additional analyses showed that differences in model outcomes were largely reduced when making changes to the structure.

Sc1. Trajectories Comparison

Aligning the models on the trajectory of HbA1c decreased the differences in outcomes between the model. This improves confidence in the estimate of the overall impact of the intervention (Table S7). The incremental difference for Brief Intervention increased when removing the HBA1c pathway, reflecting negative benefits over time for the Brief Intervention.

Sc3. Diabetes

Originally in the protocol, we’d identified a trajectory comparison as a high priority and planned to alter the SPHR model to reflect the method of estimating the trajectories used in the HC. However, it became clear that the HC model structure didn’t include a treatment pathway for HbA1c to act on the risk for diabetes. Therefore, the step was taken to remove the HbA1c trajectory from the SPHR model in order to align the trajectories structure.

A discussion with co-authors highlighted that deciding on a longitudinal dataset on which to base trajectories is a challenge due to the limited number of datasets that contain the risk factors (e.g.., BMI) and the limitations of these and therefore an additional analysis added in which we altered the SPHR model such that it was based on the ELSA dataset (same as the HC model) rather than Whitehall II dataset to examine the difference that this made to outcomes (Table S7).

Sc4. Other Conditions Comparison

Additionally, the findings support the inclusion of the additional conditions (not in both models) including lung cancer and microvascular complications. Comparisons with other models can be used to identify other conditions that can be added and the methods and data sources that can be used to do so. For example, lung cancer is not currently in the SPHR model despite evidence that health behaviours cluster and so changes in eating behaviour may impact on smoking rates (and vice versa) and therefore lung cancer (Table S7).

Table S7. Absolute QALYs and incremental QALYs of each intervention for main analysis and four alternative model structures

|  | *Absolute QALYs* | | | *Incremental QALYs* | | |
| --- | --- | --- | --- | --- | --- | --- |
|  | SPHR | HC | Percentage Difference | SPHR | HC | Percentage Difference |
| *Main analysis (after standardisation)* | | | |  |  |  |
| Simulated natural history | 11.3675 [10.5124, 12.1586] | 13.1084 [12.7644, 13.4805] | 1.7409 (15%) |  |  |  |
| Brief intervention | 11.3694 [10.5161, 12.1584] | 13.1154 [12.7644, 13.4808] | 1.7460 (15%) | 0.0019 [-0.0499, 0.0416] | 0.0078 [0.0039, 0.0125] | 0.00591 (311%) |
| 12-week intervention | 11.3923 [10.5479, 12.1737] | 13.1157 [12.7647, 13.4809] | 1.7234 (15%) | 0.0248 [-0.0024, 0.0599] | 0.0079 [0.0043, 0.0126] | -0.01695 (-68%) |
| 52-week intervention | 11.3973 [10.5663, 12.1715] | 13.1160 [12.7656, 13.4845] | 1.7187 (15%) | 0.0298 [-0.002, 0.0688] | 0.0085 [0.0048, 0.0129] | -0.0213 (-71%) |
| *Structural Sensitivity 1: SPHR with no HbA1c treatment effect* | | |  |  |  |  |
| Simulated natural history | 11.3652 [10.4815, 12.2029] | 13.1084 [12.7644, 13.4805] | 1.7432 (15%) |  |  |  |
| Brief intervention | 11.3730 [10.4728,12.1967] | 13.1154 [12.7644, 13.4808] | 1.7424 (15%) | 0.00778 [-0.0128, 0.0346] | 0.0078 [0.0039, 0.0125] | 0.00003 (0.39%) |
| 12-week intervention | 11.3799 [10.4763,12.2063] | 13.1157 [12.7647, 13.4809] | 1.7358 (15%) | 0.01469 [-0.0066, 0.0432] | 0.0079 [0.0043, 0.0126] | -0.00684 (-47%) |
| 52-week intervention | 11.3837 [10.4794,12.2080] | 13.1160 [12.7656, 13.4845] | 1.7323 (15%) | 0.01866 [-0.0055, 0.0528] | 0.0085 [0.0048, 0.0129] | -0.01016 (-54%) |
| *Structural Sensitivity 2: Both models with only CVD, Diabetes and Dementia AND no HbA1c treatment effect* | | | | | | |
| Simulated natural history | 12.7416 [12.0517, 13.1690] | 13.7603 [13.4245, 14.1369] | 1.0277 (8%) |  |  |  |
| Brief intervention | 12.7533 [12.0729, 13.1744] | 13.7706 [13.4351, 14.1468] | 1.0173 (8%) | 0.01177 [-0.0041, 0.0306] | 0.0102 [0.0053, 0.0155] | -0.00157 (-13%) |
| 12-week intervention | 12.7416 [12.0756, 13.1741] | 13.7703 [13.4338, 14.1477] | 1.0287 (8%) | 0.01331 [-0.0017, 0.0328] | 0.0100 [0.0052, 0.0147] | -0.00331 (-25%) |
| 52-week intervention | 12.7416 [12.0799, 13.1759] | 13.7711 [13.4336, 14.1499] | 1.0295 (8%) | 0.01410 [-0.0011, 0.0333] | 0.0108 [0.0049, 0.0170] | -0.0033 (-23%) |
| *Structural Sensitivity 3 3: SPHR model with risk factors trajectories based on analysis of ELSA data instead of Whitehall II without structural sensitivity A & B applied* | | | | | | |
| Simulated natural history | 11.3701 [10.4810, 12.1360] | 13.1084 [12.7644, 13.4805] | 1.7383 (15%) |  |  |  |
| Brief intervention | 11.3685 [10.4964, 12.1333] | 13.1154 [12.7644, 13.4808] | 1.7469 (15%) | -0.0016 [-0.0529, 0.0387] | 0.0078 [0.0039, 0.0125] | 0.0062 (588%) |
| 12-week intervention | 11.3958 [10.5247, 12.1620] | 13.1157 [12.7647, 13.4809] | 1.7199 (15%) | 0.0257 [-0.0003, 0.0521] | 0.0079 [0.0043, 0.0126] | 0.0178 (-69%) |
| 52-week intervention | 11.4001 [10.5288, 12.1635] | 13.1160 [12.7656, 13.4845] | 1.7159 (15%) | 0.0301 [-0.0017, 0.0626] | 0.0085 [0.0048, 0.0129] | 0.0216 (-72%) |
| *Structural Sensitivity 1,2,3: SPHR model with risk factors trajectories based on analysis of ELSA data instead of Whitehall II with structural sensitivity A & B applied* | | | | | | |
| Simulated natural history | 12.7638 [11.9646, 13.5359] | 13.1084 [12.7644, 13.4805] | 0.3442 (2.63%) |  |  |  |
| Brief intervention | 12.7479 [11.9467, 13.5267] | 13.1154 [12.7644, 13.4808] | 0.3675 (2.88%) | -0.0159 [-0.0672, 0.0185] | 0.0078 [0.0039, 0.0125] | 0.0062 (304%) |
| 12-week intervention | 12.7703 [11.9739, 13.5512] | 13.1157 [12.7647, 13.4809] | 0.3454 (2.70%) | 0.0065 [-0.0153, 0.0250] | 0.0079 [0.0043, 0.0126] | 0.0178 (18%) |
| 52-week intervention | 12.7726 [11.9833, 13.5476] | 13.1160 [12.7656, 13.4845] | 0.3434 (2.69%) | 0.0088 [-0.0163, 0.0321] | 0.0085 [0.0048, 0.0129] | 0.0216 (-4%) |

Table S8. Number of events estimated for each intervention for main analysis and four alternative model structures

|  | *Cardiovascular disease* | | *Stroke* | | *Dementia* | | *Diabetes* | |
| --- | --- | --- | --- | --- | --- | --- | --- | --- |
|  | SPHR | HC | SPHR | HC | SPHR | HC | SPHR | HC |
| *Main analysis (after standardisation)* | | | | | | | | |
| Simulated natural history | 411 | 350 | 208 | 119 | 229 | 99 | 356 | 546 |
| Brief intervention | 411 | 348 | 208 | 119 | 228 | 95 | 355 | 546 |
| 12-week intervention | 410 | 348 | 207 | 119 | 228 | 95 | 349 | 546 |
| 52-week intervention | 410 | 348 | 207 | 119 | 228 | 95 | 349 | 546 |
| *Structural Sensitivity 1: SPHR with no HbA1c treatment effect* | | | | | | | | |
| Simulated natural history | 414 | 350 | 209 | 119 | 229 | 99 | 337 | 546 |
| Brief intervention | 413 | 348 | 209 | 119 | 228 | 95 | 352 | 546 |
| 12-week intervention | 413 | 348 | 209 | 119 | 228 | 95 | 351 | 546 |
| 52-week intervention | 413 | 348 | 209 | 119 | 228 | 95 | 351 | 546 |
| *Structural Sensitivity 2: Both models with only CVD, Diabetes and Dementia AND no HbA1c treatment effect* | | | | | | | | |
| Simulated natural history | 477 | 404 | 221 | 140 | 214 | 117 | 366 | 535 |
| Brief intervention | 474 | 402 | 220 | 139 | 213 | 112 | 367 | 535 |
| 12-week intervention | 474 | 402 | 220 | 139 | 213 | 112 | 355 | 535 |
| 52-week intervention | 473 | 402 | 220 | 139 | 213 | 112 | 355 | 535 |
| *Structural Sensitivity 3 3: SPHR model with risk factors trajectories based on analysis of ELSA data instead of Whitehall II without structural sensitivity A & B applied* | | | | | | | | |
| Simulated natural history | 391 | 350 | 196 | 119 | 245 | 99 | 357 | 546 |
| Brief intervention | 390 | 348 | 196 | 119 | 245 | 95 | 364 | 546 |
| 12-week intervention | 389 | 348 | 196 | 119 | 245 | 95 | 357 | 546 |
| 52-week intervention | 389 | 348 | 196 | 119 | 245 | 95 | 357 | 546 |
| *Structural Sensitivity 1,2,3: SPHR model with risk factors trajectories based on analysis of ELSA data instead of Whitehall II with structural sensitivity A & B applied* | | | | | | | | |
| Simulated natural history | 463 | 404 | 211 | 140 | 214 | 117 | 366 | 535 |
| Brief intervention | 463 | 402 | 211 | 139 | 213 | 112 | 367 | 535 |
| 12-week intervention | 462 | 402 | 211 | 139 | 213 | 112 | 355 | 535 |
| 52-week intervention | 463 | 402 | 211 | 139 | 213 | 112 | 355 | 535 |

Model Adaptations not implemented

S2 CVD Risk Comparison

For scenario two, we had planned to compare methods of CVD risk factors. Given that there were small differences in the CVD outcomes; we prioritised examining the impact of including different conditions.

Sc5. Opportunistic Diagnosis and Treatment Comparison

Given that there was small difference in the CVD outcomes; we prioritised examining the impact of including different conditions. The treatment effects for cholesterol and systolic blood pressure were not statistically significant for the intervention, so it was concluded that this model adaptation would not be useful for this application of the model.

Sc6. Dementia Risk Comparison

Previous analyses of the SPHR model concluded that the inclusion of Dementia into the model had a modest impact on health economic outcomes. Although there was a notable difference in absolute dementia incidence predicted across models, it was concluded that the differences were unlikely to be driving the differences in incremental outcomes between model outcome once discounting was taken into account.

Sc7.Utilities Comparison

The structure coding of utility decrements made this model modification difficult to implement. This scenario would have provided a very interesting examination of potential affects of additive versus multiplicative approaches to utility decrements, but this was not feasible within this project.

Conclusions

The following conclusions were drawn from our model comparison.

- - 1. Incremental outcomes were impacted by the presence of an HbA1c trajectory emphasising the importance of representing the trajectories of all metabolic risk factors, potentially affected, directly or indirectly, by an intervention. This structural modification produced the strongest change in incremental QALYs for the 12 week WW and 52 week WW.
    2. Including a wide range of conditions that are likely to be directly or indirectly affected by the intervention is important to capture all benefits and costs and measure the full incremental benefits.
    3. The dataset on which the baseline population is based doesn’t make a large difference to outcomes, as long as it is representative and includes the characteristics required for the model (including EQ5D).
    4. Risk factors trajectories based on ELSA rather than Whitehall II did not have large impacts on QALYs, except whether the simulated trajectory for the control produced lower average BMI than the Brief Intervention arm.

Supplementary Appendix D: Proposed recommendation for Cross-model validation

***Proposed Recommendation R1 – Select a model where there is full access to code, data and technical descriptions, and opportunity to collaborate with model developers***

Access to the model code is ideal for an in-depth understanding of the models and ability to conduct adaptations required for standardisation and further alignment of models.

***Proposed Recommendation R2 – Use a protocol to inform the iterative validation process***

A protocol should be used to describe the models, document model differences and propose potential model modifications. We recommend using the protocol as a guide for the validation process developed and approved by all collaborators prior to model comparison.

***Proposed Recommendation R3 – Calculate the impact of standardising model setup***

We recommend standardising the model setup before their results are compared. Stepwise documentation of the standardisation steps, and the impact of each step on the model outputs, can help to understand how sensitive the model is to specifications. This can help to motivate further model adaptations and new model development.

***Proposed Recommendation R4 – Observe difference across a broad range of health economic outcomes and intermediate outcomes***

We recommend conducting an in-depth assessment of consistency across multiple outcomes (including aggregates outcomes, incremental values, intermediate outcomes such as diagnoses of health conditions, and outcomes for relevant subgroups) and uncertainty analysis, with an aim to understand the differences between the models.

***Proposed Recommendation R5 – Conduct iterative adjustments to make model more similar in terms of parameters and structure to identify drivers of differences***

Running iterations of the models in which the model structures are made more similar can increase our understanding of how differences in model structure contribute to the differences in model outcomes. The options for model adaptations should be pre-specified in the protocol, but the choice of which changes to make will be informed by the outcomes of the standardisation steps, the initial model comparison, discussions with the model developers and practical considerations of which aspects of the model can be easily modified.

***Proposed Recommendation R6 – Make validation reports and results publicly available using open science principles to inform decision making and future model development***

The results of the model comparison should be reported, including details of the comparisons made and impact of model structure on outcomes, so that it can be used to inform decision making and future modelling decisions.

Figure S1. Proposed recommendations and application of cross-model validation of public health microsimulation models

*
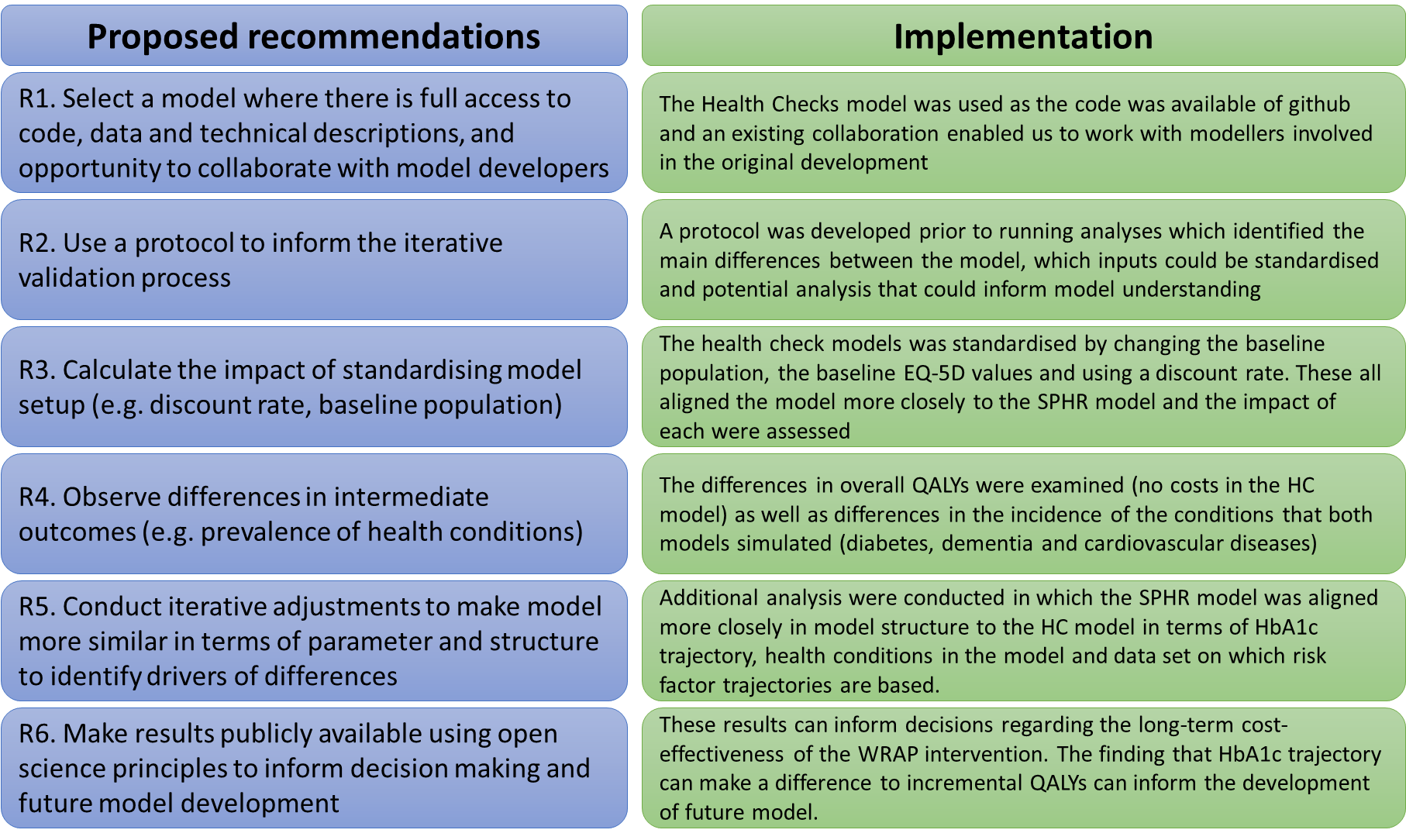
*

*Legend: Proposed recommendations that researchers can follow to compare models for cross-model validation based on the authors experience of conducting this process, alongside how these steps are exemplified in the specific cross-model validation reported in the main paper.*

References

1 Mindell J, Biddulph JP, Hirani V, et al. Cohort profile: the health survey for England. *International journal of epidemiology*. 2012;41(6):1585-1593.

2 Marmot M, Brunner E. Cohort profile: the Whitehall II study. *International journal of epidemiology*. 2005;34(2):251-256.

3 Breeze P, Squires H, Chilcott J, et al. A statistical model to describe longitudinal and correlated metabolic risk factors: the Whitehall II prospective study. *Journal of Public Health*. 2016;38(4):679-687.
